# Monocomponent Photoinitiators based on Benzophenone-Carbazole Structure for LED Photoinitiating Systems and Application on 3D Printing

**DOI:** 10.3390/polym12061394

**Published:** 2020-06-22

**Authors:** Shaohui Liu, Hong Chen, Yijun Zhang, Ke Sun, Yangyang Xu, Fabrice Morlet-Savary, Bernadette Graff, Guillaume Noirbent, Corentin Pigot, Damien Brunel, Malek Nechab, Didier Gigmes, Pu Xiao, Frédéric Dumur, Jacques Lalevée

**Affiliations:** 1 Université de Haute-Alsace, CNRS, IS2M UMR 7361, F-68100 Mulhouse, France; shaohui.liu@uha.fr (S.L.); hong.chen@uha.fr (H.C.); yijun.zhang@uha.fr (Y.Z.); ke.sun@uha.fr (K.S.); ahutxyy@163.com (Y.X.); fabrice.morlet-savary@uha.fr (F.M.-S.); bernadette.graff@uha.fr (B.G.); 2 Université de Strasbourg, Grand Est, 67200 Strasbourg, France; 3 Aix Marseille Univ, CNRS, ICR UMR 7273, F-13397 Marseille, France; guillaume.noirbent@outlook.fr (G.N.); corentin.pigot@univ-amu.fr (C.P.); damien.brunel@univ-amu.fr (D.B.); malek.nechab@univ-amu.fr (M.N.); didier.gigmes@univ-amu.fr (D.G.); 4 Research School of Chemistry, Australian National University, Canberra, ACT 2601, Australia

**Keywords:** monocomponent photoinitiator, carbazole, 3D printing, benzophenone

## Abstract

In this article, different substituents (benzoyl, acetyl, styryl) are introduced onto the carbazole scaffold to obtain 8 novel carbazole derivatives. Interestingly, a benzoyl substituent, connected to a carbazole group, could form a benzophenone moiety, which composes a monocomponent Type II benzophenone-carbazole photoinitiator (PI). The synergetic effect of the benzophenone moiety and the amine in the carbazole moiety is expected to produce high performance photoinitiating systems (PISs) for the free radical photopolymerization (FRP). For different substituents, clear effects on the light absorption properties are demonstrated using UV-Visible absorption spectroscopy. Benzophenone-carbazole PIs can initiate the FRP of acrylates alone (monocomponent Type II photoinitiator behavior). In addition, fast polymerization rates and high function conversions of acrylate are observed when an amine and/or an iodonium salt are added in systems. Benzophenone-carbazole PIs have good efficiencies in cationic photopolymerization (CP) upon LED @ 365 nm irradiation in the presence of iodonium salt. In contrast, other PIs without synergetic effect demonstrate unsatisfied photopolymerization profiles in the same conditions. The best PIS identified for the free radical photopolymerization were used in three-dimensional (3D) printing. Steady state photolysis and fluorescence quenching experiments were carried out to investigate the reactivity and the photochemistry and photophysical properties of PIs. The free radicals, generated from the studied PISs, are detected by the electron spin resonance - spin trapping technique. The proposed chemical mechanisms are provided and the structure/reactivity/efficiency relationships are also discussed. All the results showed that the benzophenone-carbazole PIs have a good application potential, and this work provides a rational design route for PI molecules. Remarkably, BPC2-BPC4, C6, C8 were never synthetized before; therefore, 5 of the 8 compounds are completely new.

## 1. Introduction

Photopolymerization has attracted broad attention from both the academic and industrial communities, due to significant advantages, including a wide range of applications, high efficiency and no volatile organic compounds emissions [1,2,3,4,5,6]. In recent years, light-emitting diodes (LEDs) as irradiation sources are used more and more widely in photopolymerization [7,8,9]. Compared to UV lamp and UV lasers, LEDs exhibit a higher security, less heat generation, as well as no ozone release in runtime [10,11,12]. LEDs demonstrate tremendous potential as an alternative to the traditional UV irradiation sources. Three-dimensional (3D) printing by photopolymerization provides a unique approach to convert liquid (or viscous) resins into solids displaying the desired shape upon light irradiation. Parallel to this, three-dimensional printing has been applied in a broad range of fields, including the elaboration of dental materials, various equipment in the biomedical fields or in the robotic area. Due to its good forming ability and convenient operation, 3D printing using photopolymerization technique has gain increased attention [13,14,15].

Nowadays, exploring the new photoinitiating systems (PISs) matching LEDs are becoming important and urgent [16,17]. As a crucial role in PISs, the photoinitiator (PI) could generate active species under irradiation to initiate cationic and/or free radical polymerizations. Compared to the traditional Type I cleavage systems and Type II H abstraction systems, dye-photosensitized systems, which can extensively use LEDs and can be excited under long-wavelength light, are attracting more intention in photopolymerization [18,19]. Due to its good optical properties, carbazole has been frequently used to design functional PIs [20,21]. Moreover, carbazole is an excellent electron donor that can be oxidized at low potential. In order to obtain excellent light absorption properties, electron-donating moiety and electron-accepting moiety can be connected to the carbazole core, in order to elaborate push-pull structure dyes [22]. Indeed, numerous carbazole derivatives as PIs show a good performance in photopolymerization [23,24,25]. Benzophenone (BP) is a commercial Type II PI, which can initiate the free radical photopolymerization, but only in the presence of co-initiators (e.g., amines) under UV irradiation. Due to the high photoinitiation efficiency of BP, many structures comprising the BP moiety were studied as exemplified with the design of polymeric PIs or the elaboration of monocomponent Type II PI [26,27,28]. However, many of these reported systems are still used as traditional UV PISs [29,30] using Hg lamps. In recent years, the design of visible light BP-based dyes has been extensively studied and benzophenone-pyrene, benzophenone-naphthalimide and thioxanthone-carbazole compounds were reported as efficient PIs under LED irradiation [31,32,33,34]. In addition, a bifunctional benzophenone-carbazole photoinitiator has been recently reported by our group for FRP, and the results show that the photoinitiator exhibits excellent light absorption properties and photoinitiation ability [35]. 

In present work, benzoyl substituents were introduced onto the carbazole core and the combination within a unique structure of a benzophenone group, and an amine included in the carbazole moiety, offer a unique opportunity in elucidating monocomponent Type II PIS. To demonstrate the benefits of this unique combination, PIs, based on carbazoles connected to acetyl or styryl substituents, were prepared for comparison. The free radical photopolymerization will be studied in the presence of carbazole derivatives without additives (e.g., co-initiators). Also, the co-initiators iodonium salt or/and amine are added into the PISs to generate the active species for cationic photopolymerization and free radical photopolymerization. The photoinitiation ability of the carbazole derivatives was evaluated with benchmark acrylate and epoxy monomers. The best PIS identified during the free-radical polymerization of acrylate was applied for the generation of 3D patterns. In addition, the photochemical mechanisms were investigated by the steady state photolysis, fluorescence quenching and electron spin resonance spin-trapping approaches. 

## 2. Materials and Methods

### 2.1. Materials

**Scheme 1:**
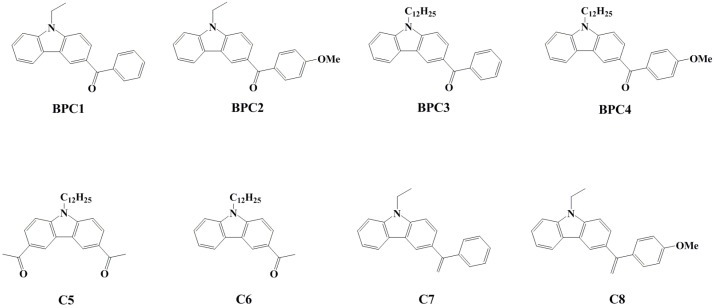
The structures of carbazole derivatives in this study.

The structures of the different carbazole derivatives are shown in Scheme 1. The synthesis of these new photoinitiators is presented in detail in the supporting information. Remarkably, BPC2-BPC4, C6, C8 were never synthetized before; therefore, 5 of the 8 compounds are completely new. 

**Scheme 2:**
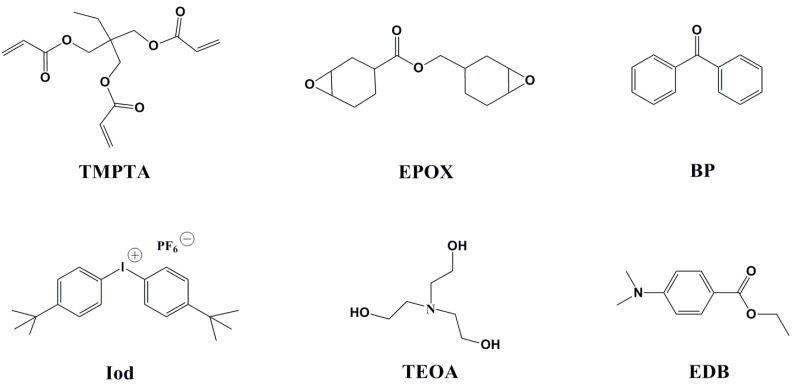
Chemical structures of monomers and additives used in this study.

Bis(4-*tert*-butylphenyl)iodonium hexafluorophosphate (Iod), ethyl 4-(dimethylamino)benzoate (EDB) and benzophenone (BP) were obtained from Lambson Ltd. (Wetherby, United Kingdom). Phenyl-*N*-*tert*-butylnitrone (PBN) used as the free radical trapping agent and triethanolamine (TEOA) were purchased from TCI-Europe (Paris, France). Trimethylolpropane triacrylate (TMPTA) and (3,4-epoxycyclohexane)methyl 3,4-epoxycyclohexylcarboxylate (EPOX) were obtained from Allnex (Ivry sur Seine, France) and used as benchmark monomers for the free radical photopolymerization, and the cationic photopolymerization, respectively. Chemical structures of some compounds used in this study are given in the Scheme 2.

### 2.2. UV-Vis Absorption Spectra

The UV−visible absorption properties of the different PISs were studied using a JASCO V730 spectrophotometer (JASCO, Lisses, France).

### 2.3. Computational Procedure

Molecular orbital calculations were carried out with the Gaussian 03 suite of programs. Absorption properties of the carbazole derivatives were calculated with the time-dependent density functional theory at the MPW1PW91/6-31G* level of theory on the relaxed geometries calculated at the UB3LYP/6-31G* level of theory.

### 2.4. Cationic Photopolymerization and Free Radical Photopolymerization

In this study, the different carbazole derivatives, EDB, Iod, TMPTA or EPOX were added into a glass bottle. The weight percent of the PI or co-initiators was calculated from the monomer content (e.g., PI/Iod (0.5%/1% *w/w*) = 0.005g PI, 0.01g Iod, 1g monomer). The mixture was stirred for 24 h in a dark environment. Photosensitive resins (∼25 μm of thickness) were irradiated under air for the cationic photopolymerization of EPOX. The conversion of the epoxy group was continuously monitored by Real-Time Fourier Transformed Infrared Spectroscopy (RT-FTIR, JASCO FTIR 4100, JASCO, Lisses, France) at about 790 cm^−1^. The free radical photopolymerization of TMPTA was carried out in laminate (between two polypropylene films to reduce the O_2_ inhibition). The evaluation of the acrylate characteristic peak was continuously followed by RT-FTIR at around 1630 cm^−1^. A LED@365 nm was used as the irradiation sources. Light intensity at the sample surface was I_0_ ≈ 40 mW cm^−2^ and the irradiation starts at *t* = 10 s.

### 2.5. 3D Printing Experiments

A laser diode (Thorlabs, Exceter, United Kingdom) (spot size around 50 μm) was used to obtain the specific 3D patterns on the basis of the photosensitive resins under air. The obtained 3D patterns were analyzed by a numerical optical microscope (OLYMPUS DSX-HRSU).

### 2.6. Fluorescence Experiments

Fluorescence properties of the different compounds were studied using the JASCO FP-6200 spectrometer (Jasco, Lisses, France).

### 2.7. Electron Spin Resonance - Spin Trapping (ESR-ST)

ESR-ST experiments were carried out using an X-band spectrometer (Bruker EMX-Plus, JASCO, Lisses, France)). Phenyl-*N*-*tert*-butylnitrone (PBN) was used as the free radical trapping agent. N_2_ saturated *tert*-Butylbenzene solutions were irradiated upon LED@365 nm in room temperature. Furthermore, the ESR spectra simulations were carried out with the PEST WINSIM program.

## 3. Results

### 3.1. Light Absorption Properties and Molecular Orbital Calculations on the Optimized Geometries of the Carbazole Derivatives

**Figure 1:**
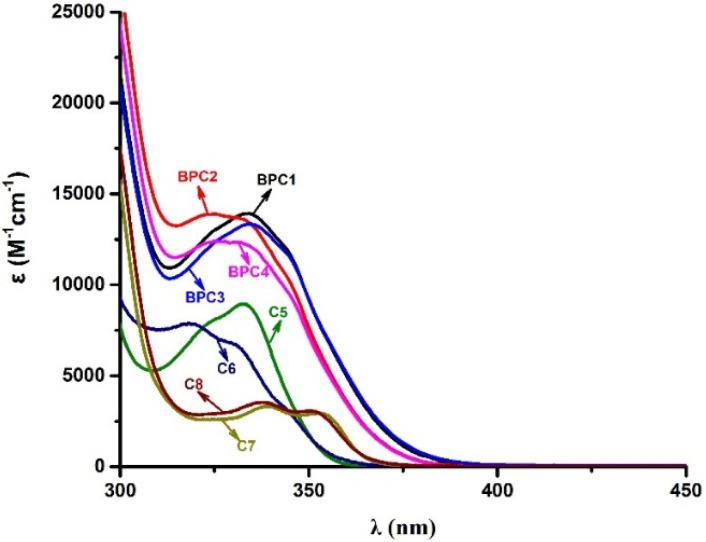
UV-visible absorption spectra of the carbazole derivatives in acetonitrile.

**Figure 2:**
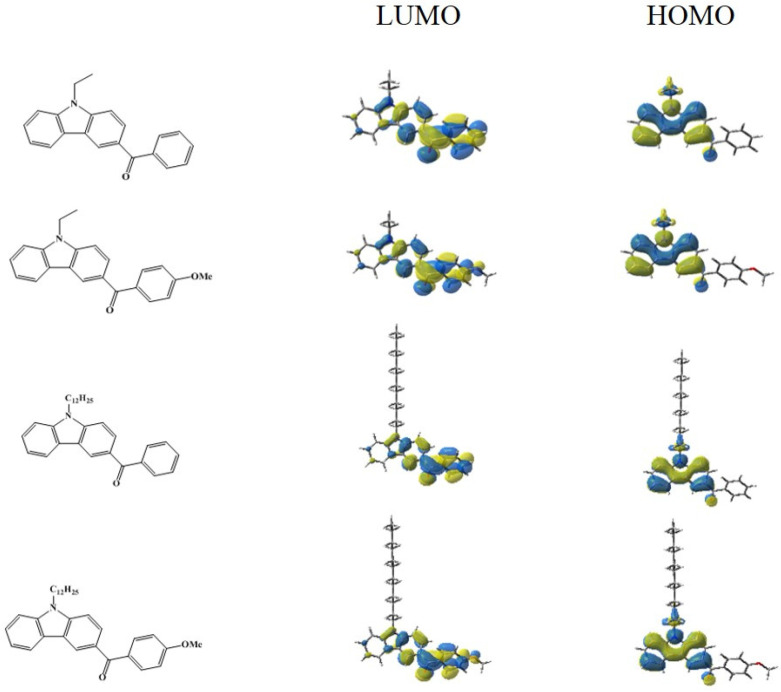
Highest occupied molecular orbital (HOMO) and lowest unoccupied molecular orbital (LUMO) of BPC1-BPC4 calculated at the B3LYP/6-31G* level of theory.

UV-Vis absorption spectra of the different carbazole derivatives were recorded in acetonitrile the spectra are given in Figure 1. Their maximum absorption wavelengths (λ_max_), molar absorption coefficients at maximum absorption wavelength (ε_max_) and molar absorption coefficients at 365 nm (ε_365 nm_) are summarized in the Table 1. The results show that the maximum absorption wavelengths of all carbazole derivatives are located in the UV range. Compared to C5–C8, the benzophenone-carbazole compounds BPC1-BPC4 showed excellent absorption properties. Especially, for BPC1, a high molar absorption coefficient ε_max_ = 13910 M^−1^cm^−1^ and an absorption maximum λ_max_ = 334 nm are observed. Molar absorption coefficients at 365 nm of BPC1-BPC4 are also higher than those of C5-C8. Molecular orbital calculations on the optimized geometries of BPC1-BPC4 are depicted in Figure 2. The charge transfer transition between highest occupied molecular orbital (HOMO) and the lowest unoccupied molecular orbital (LUMO) exhibits a π-π* character for BPC1-BPC4. The HOMO energy level is located on the electron-donating carbazole-formyl group and the LUMO level is mostly located onto the electron-accepting benzophenone group.

### 3.2. Cationic Photopolymerization and Free Radical Photopolymerization

**Figure 3:**
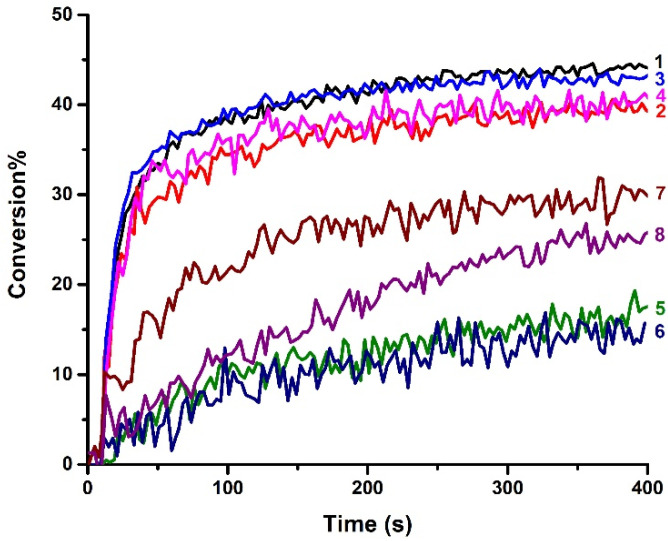
Photopolymerization profiles of EPOX (epoxy function conversion vs. irradiation time) upon LED@365 nm irradiation under air in the presence of (1) BPC1/Iod (0.5%/1% *w/w*); (2) BPC2/Iod (0.5%/1% *w/w*); (3) BPC3/Iod (0.5%/1% *w/w*); (4) BPC4/Iod (0.5%/1% *w/w*); (5) C5/Iod (1%/1% *w/w*); (6) C6/Iod (0.5%/1% *w/w*); (7) C7/Iod (0.5%/1% *w/w*); (8) C8/Iod (0.5%/1% *w/w*). The irradiation starts from t = 10 s.

The cationic photopolymerization of EPOX in the presence of the two-component PISs PI/Iod (0.5%/1% *w/w*) was carried out under air upon a LED@365 nm irradiation. The polymerization profiles are shown in Figure 3 and the final function conversions of epoxy group for EPOX are shown in Table 2. It is obvious that the benzophenone-carbazole PIs BPC1-BPC4 based two-component PISs demonstrate fast polymerization rates and high final function conversions. BPC1/Iod system shows the highest epoxy function conversion 44%, and the conversions initiated by BPC2-BPC3/Iod systems are all close to 40%. Relatively, C5-C8/Iod systems displayed worse performances than the BPC1-BPC4/Iod systems (Figure 3, curves 5–8 vs. curves 1–4). Due to their high molar absorption coefficients, there is an efficient utilization with light for BPC1-BPC4 at 365 nm. The results obtained during the cationic photopolymerization of EPOX are in accordance with their light absorption properties.

**Figure 4:**
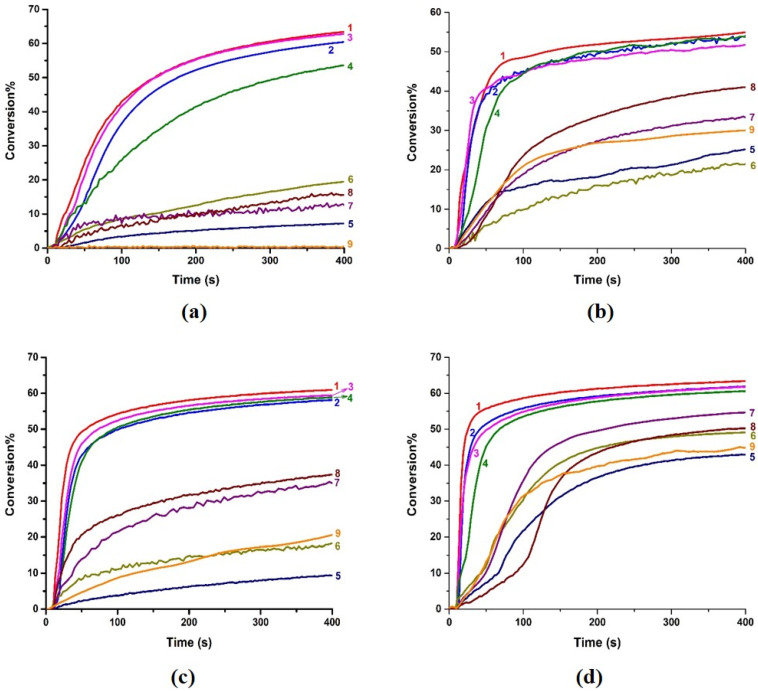
Photopolymerization profiles of TMPTA (acrylate function conversion vs. irradiation time) in laminate upon LED@365 nm irradiation in the presence of (**a**) PI alone (1.5%, w), (**b**) PI/EDB (0.5%/1%, *w/w*), (**c**) PI/Iod (0.5%/1%, *w/w*), (**d**) PI/EDB/Iod (0.5%/1%/1%, *w/w/w*). (curve 1—BPC1-based PISs, curve 2—BPC2-based PISs, curve 3—BPC3-based PISs, curve 4—BPC4-based PISs, curve 5—C5-based PISs, curve 6—C6-based PISs, curve 7—C7-based PISs, curve 8—C8-based PISs, curve 9—BP-based PISs); the irradiation starts from *t* = 10 s.

The free radical photopolymerization of TMPTA was studied in the presence of PI alone, PI/EDB, PI/Iod and PI/EDB/Iod respectively. The polymerization was carried out upon irradiation with a LED@365 nm irradiation in laminate. Typically, for BPC1-BPC4, there exist monocomponent Type II PISs in their chemical structures. It is possible that the H abstraction reaction occurs easily between the benzophenone and the carbazole moiety. Therefore, the free radical photopolymerization was investigated when the carbazole derivative (BPC1-BPC4) was used alone as PI. Interestingly, BPC1-BPC4 alone can initiate the polymerization of TMPTA effectively under the light irradiation, as shown in Figure 4a. The function conversion of TMPTA in the presence of BPC1 alone can reach 64% which is the highest value among the series. There are high acrylate function conversions for BPC2-BPC4 also. While C5-C8 lead to very low function conversions (below 20%). Obviously, it is certain that benzophenone-carbazole PIs can initiate the polymerization of acrylate monomers effectively without external co-initiator (e.g., EDB). It could be ascribed to the H abstraction from the carbon atom adjacent to N of the carbazole unit (see below for the chemical mechanisms). No polymerization is observed for BP alone (Figure 4a, curve 9).

EDB as co-initiator was added to study the photoinitiation ability of PIs. The polymerization profiles of TMPTA in the presence of PI/EDB systems are given in Figure 4b. With the addition of EDB, the polymerization rates of PI/EDB systems are faster than those of PI alone systems (higher polymerization rates). The function conversion of TMPTA initiated by BP/EDB as reference system is only 30% (Figure 4b, curve 9). It confirms that upon LED@365 nm irradiation the BP moiety in BPC1-BPC4 structures also keep a high capacity for H abstraction. BPC1-BPC4 systems showed good performance, especially BPC1 which resulted in the highest function conversion (55%) of TMPTA. The photopolymerization performance of C5-C8 is unsatisfied. In other words, the functional benzophenone group has a positive effect on the reaction activity.

The polymerization profiles of PI/Iod systems are shown in Figure 4c. BPC1-BPC4/Iod systems exhibit excellent efficiencies upon LED@365 nm irradiation. It is worth mentioning that, besides the interactions between benzophenone-carbazole compounds (as photosensitizers) and Iod, there are still interactions between two molecules of benzophenone-carbazole compounds (as monocomponent Type II PIs) to promote free radical photopolymerization. C5-C8/Iod and BP/Iod systems demonstrate slow polymerization rates and low function conversions. The same trend also appears for the PI/EDB/Iod systems in Figure 4d. Furthermore, the polymerization rates and function conversions of TMPTA initiated by PI/EDB/Iod systems are better than PI/EDB and PI/Iod systems. All of the final acrylate function conversions of TMPTA are summarized in Table 2.

### 3.3. Steady State Photolysis

**Figure 5:**
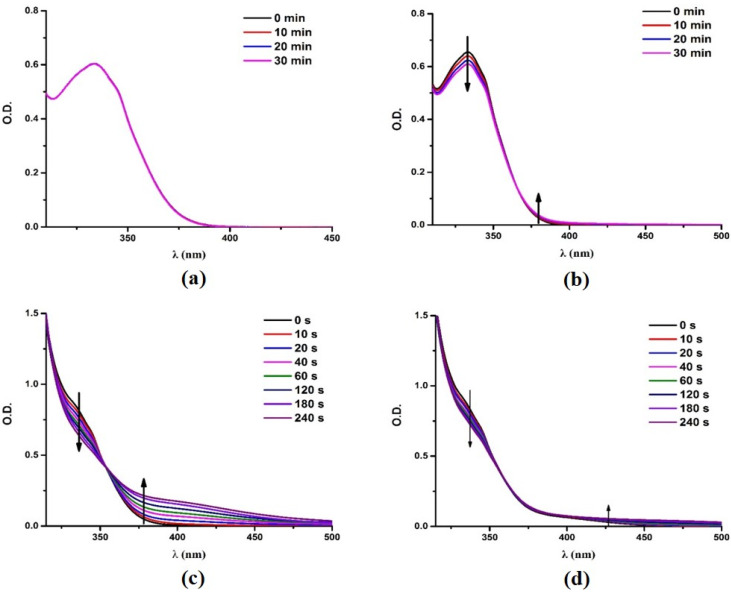
Photolysis of (**a**) BPC1 alone; (**b**) BPC1/TEOA; (**c**) BPC1/Iod; (**d**) BPC1/TEOA/Iod upon LED@375 nm irradiation in acetonitrile ([Iod] = 0.01 M, [TEOA] = 0.01 M).

To study the activity of PIs, UV-Vis spectra of the different PISs in acetonitrile upon LED@375 nm irradiation, were recorded at different times. The absorption peak of PIs partly overlaps with that of EDB so that triethanolamine (TEOA), which is also a tertiary amine, was used instead of EDB in this study. The photolysis of BPC1-based systems is shown in Figure 5. As for BPC1 alone (Figure 5a), the spectra remained mainly unchanged from 0 min to 30 min irradiation, which can be ascribed to the low concentration of BPC1 in solution. In this case, the interaction between the benzophenone moiety and the carbazole moiety of two molecules of BPC1 was week. The absorbance of BPC1/TEOA system decreased slowly in 30 min (Figure 5b). It could be attributed to the H abstraction occurring between the benzophenone moiety in BPC1 and the amine in TEOA. Interactions of BPC1/Iod (Figure 5c) and BPC1/TEOA/Iod (Figure 5d) were quite fast during 240 s light irradiation. For BPC1/Iod system, the change of absorption spectrum was obvious, and the results demonstrated that there were effective interactions in the excited states. In addition, the isosbestic points showed that the reaction of BPC1/EDB/Iod system had no by-products in the process of photolysis. The consumption rate of BPC1 in BPC1/TEOA/Iod system was slower than BPC1/Iod system. It is probable that there was a photoredox catalytic cycle in the three-component system regenerating BPC1.

**Figure 6:**
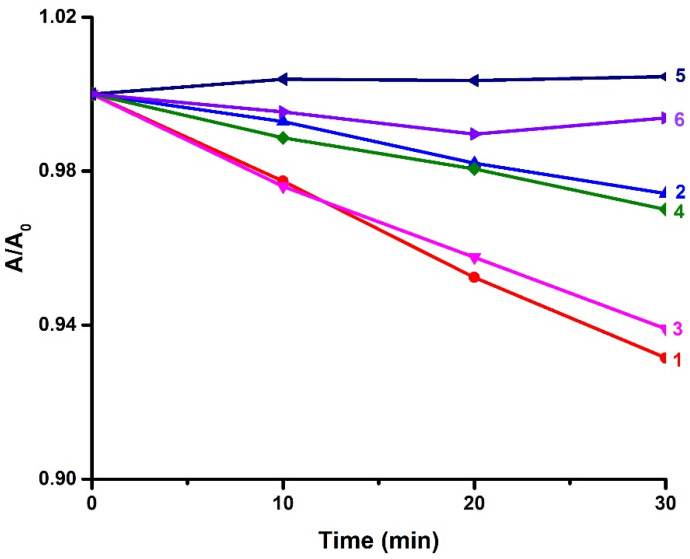
Photolysis kinetics of PI/TEOA systems upon LED@375 nm irradiation in acetonitrile (**1**) BPC1/TEOA, (**2**) BPC2/TEOA, (**3**) BPC3/TEOA, (**4**) BPC4/TEOA, (**5**) C5/TEOA, (**6**) C7/TEOA. (*A_0_* absorbance of maximum wavelength at 0 min, *A* absorbance of maximum wavelength at *t* min.).

As shown in Figure 6, the photolysis kinetics of PI/TEOA systems revealed the consumption rates of PI. The photolysis of BPC2-BPC4/TEOA, C5/TEOA and C7/TEOA are given in Figure S1. The consumption rates of BPC1-BPC4 were faster than C5 and C7. The results showed that the presence of the benzophenone moiety in BPC1-BPC4 structures promoted the reaction between PI and amine. The photolysis demonstrated the benzophenone-carbazole PIs had high reactivity. 

### 3.4. Fluorescence Quenching Experiments

**Figure 7:**
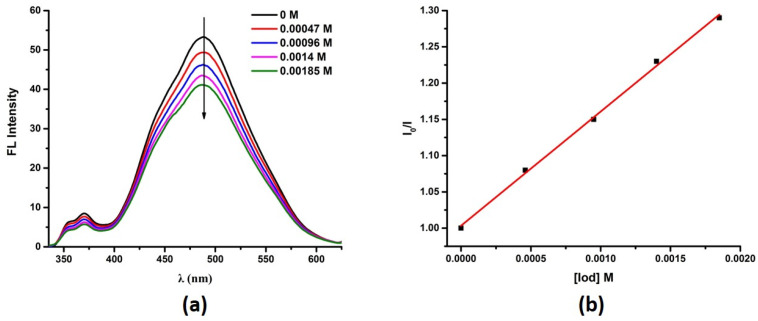
(**a**) Fluorescence quenching of BPC1 by Iod in acetonitrile; (**b**) Stern-Volmer treatment for BPC1/Iod fluorescence quenching.

The fluorescent properties can evidence the interaction capacity of PIs with additives in the excited singlet state. Fluorescence quenching experiments were carried out in acetonitrile. The result of BPC1/Iod system is shown in Figure 7a and the BPC2-BPC4/Iod systems are given in Figure S2. The K_sv_ Stern-Volmer coefficients were acquired by Figure 7b and the electron transfer quantum yields in the excited singlet state Φ_et_(s1) were calculated. The parameters were listed in Table 3. The high electron transfer quantum yields implied that electron transfer occurred easily between benzophenone-carbazole PIs and Iod [36]. The fluorescence quenching experiments of BPC1-BPC4/EDB systems are given in Figure S3. The results showed that the addition of EDB reduced the fluorescence intensity of BPC1-BPC4, therefore the electron transfer also occurred effectively from EDB to benzophenone-carbazole PIs in the excited singlet state. The shape change of fluorescence emission peaks may be ascribed to the generation of charge transfer complex [37].

## 4. Discussion

### 4.1. Proposed Chemical Mechanisms

**Scheme 3:**
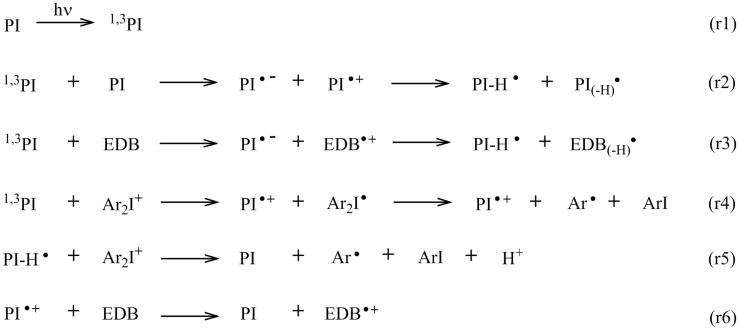
The proposed mechanism of the photoinitiating systems in this study.

**Figure 8:**
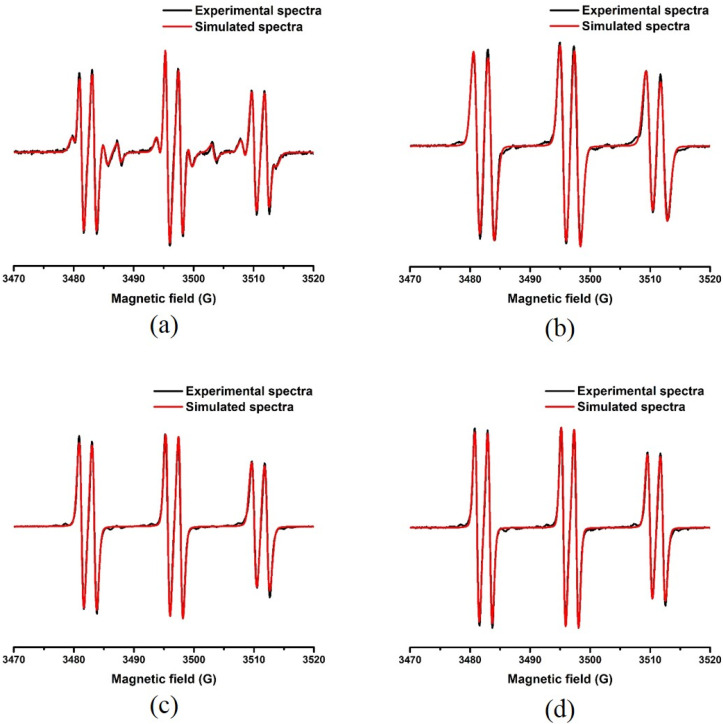
ESR-ST spectra of the radical adducts obtained in the presence of (**a**) BPC1; (**b**) BPC/EDB; (**c**) BPC1/Iod; (**d**) BPC1/EDB/Iod upon LED@365 nm irradiation in *tert*-butylbenzene.

Based on the steady state photolysis and fluorescence quenching experiments, the proposed mechanism for BPC1-BPC4 is shown in Scheme 3. Furthermore, ESR-ST experiments were carried out to investigate the generation of free radicals in the different PISs. Under irradiation, PI transforms from ground state to excited state (Scheme 3, r1). The typical H abstraction occurs from C atom adjacent to N of carbazole moiety and it is also observed in thioxanthone-carbazole compound [34]. The PI_(−H)_**˙** radical is a kind of active species for free radical polymerization. When BPC1 was alone in the system, the hyperfine coupling constants of a_N_ = 14.3 G and a_H_ = 1.9 G were obtained (Figure 8a). It is considered as PI_(−H)_**˙**/PBN radical adduct [38]. The mechanism is shown in the reaction (Scheme 3, r2).

The hyperfine coupling constants of PBN radical adducts for BPC1/EDB system are a_N_ = 14.4 G and a_H_ = 2.3 G (Figure 8b), and it is in agreement with EDB_(−H)_**˙** radical [39]. Under irradiation, BPC1 excited states interact with EDB. Radicals are generated through electron transfer and H abstraction reaction between BPC1 and EDB. EDB_(−H)_**˙** is regards as efficient active species for free radical polymerization (Scheme 3, r3). The results of steady state photolysis and fluorescence quenching experiment show that BPC1 has a high reactivity with EDB. H abstraction reaction could occur more easily from EDB than bimolecular BPC1, so the addition of EDB promotes the polymerization rate in BPC1/EDB system.

In BPC1/Iod system, hyperfine coupling constants are a_N_ = 14.3 G and a_H_ = 2.1 G (Figure 8c) typical for the Ar**˙**/PBN radical adduct [40]. The electron transfer from BPC1 to Iod generating aryl radical Ar**˙** and cation PI**˙^+^** (Scheme 3, r4) which are considered as the initiating species for the radical polymerization, and the cationic polymerization, respectively. The hyperfine coupling constants a_N_ = 14.3 G and a_H_ = 2.1 G were obtained in BPC1/EDB/Iod system (Figure 8d) which proved the presence of Ar**˙**. Associated with the photolysis of BPC1/Iod and BPC1/EDB/Iod, the photoredox catalytic cycle was proposed in three-component PI/EDB/Iod system (Scheme 3, r5 and r6). The regeneration of PI speeds up the photopolymerization and slows down the consumption of PI in the photolysis experiments [41,42].

### 4.2. Structure/Reactivity/Efficiency Relationship

For all the carbazole derivatives, the benzophenone-carbazole combining structure enhances the light absorption properties. BPC1-BPC4 based PISs exhibited better photoinitiation efficiency in free radical photopolymerization and cationic photopolymerization than others. Besides the high molar absorption coefficients, the benzophenone moiety made it easy to accept electron, meanwhile it had no effect on the electron-donating ability of the carbazole moiety. The methoxy group in BPC2 and BPC4 produced a blue-shift of the absorption spectrum and low molar absorption coefficients at 365 nm. So BPC1 and BPC3 showed a better efficiency than BPC2 and BPC4 respectively. The difference between BPC1 and BPC3 was the length of the alkyl chain attached to the carbazole moiety. BPC3 had a long carbon chain attached to N, and the steric hindrance exhibited an effect on the approach of aminoalkyl radicals to monomers. Therefore, the photoinitiation efficiency of BPC3 and BPC4 can be worse than BPC1, and BPC2, respectively.

### 4.3. 3D Printing Experiment Based on BPC1/EDB/Iod System

**Figure 9:**
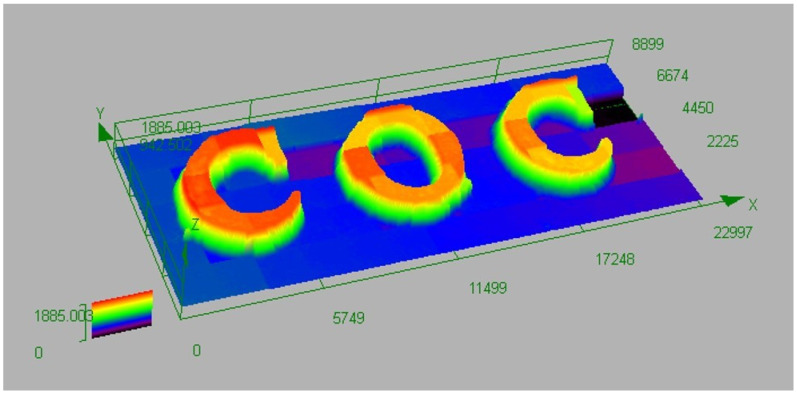
The pattern “COC” was observed by numerical optical microscopy.

The three-component BPC1/EDB/Iod system with the best performance for free radical photopolymerization of TMPTA was applied in the laser write of 3D patterns. The experiment was carried out using a computer-controlled laser diode at 405 nm and the samples were observed by numerical optical microscope. The photopolymerization profile of TMPTA upon 405 nm irradiation in the presence of BPC1/EDB/Iod was given in Figure S4. As shown in Figure 9, the thickness of the pattern “COC” was up to ~1900 nm and it showed an excellent spatial resolution (<60 μm).

## 5. Conclusions

In this work, for BPC1-BPC4, the benzophenone-carbazole combining structure not only strengthened the light absorption but also formed a monocomponent Type II PISs. As a result, BPC1-BPC4 exhibited better performance in cationic polymerization and free radical photopolymerization than C5-C8 upon LED @365 nm. Interestingly, there were high function conversions of acrylate in the presence of BPC1-BPC4 alone because of the monocomponent Type II PI behavior. When EDB and Iod were added to the systems, the polymerization rates were improved greatly. The BPC1/EDB/Iod system was also applied on 3D printing, and the 3D pattern showed good profiles. The proposed mechanism was discussed through steady state photolysis, fluorescence quenching and ESR-ST techniques. The development of new structures of PIs based on carbazole scaffold is currently under way.

## Figures and Tables

**Table 1:** Light absorption properties of the carbazole derivatives.

PI	λ_max_/nm	ε_max_/M^−1^cm^−1^	ε_365 nm_/M^−1^cm^−1^
BPC1	334	13,910	3270
BPC2	325	13,900	2210
BPC3	334	13,350	3460
BPC4	325	12,400	2170
C5	332	8590	70
C6	318	7880	130
C7	339	3320	500
C8	337	3540	540

**Table 2:** Final epoxy function conversion for EPOX and acrylate function conversion for TMPTA upon LED@365 nm irradiation at *t* = 400 s.

PISs	EPOX/%	TMPTA/%			
	PI/Iod	PI	PI/EDB	PI/Iod	PI/EDB/Iod
BPC1	44	64	55	61	63
BPC2	40	60	54	58	62
BPC3	43	63	52	59	62
BPC4	41	54	54	59	61
C5	18	7	25	9	43
C6	16	20	21	18	49
C7	29	12	33	35	55
C8	25	16	41	37	50
BP	—	0	30	21	45

**Table 3:** Some parameters of BPC1-BPC4. K_SV_ (M^−1^) Stern-Volmer coefficients for PI/Iod systems, Φ_et_(s1) PI/Iod electron transfer quantum yields in the excited singlet state.

	BPC1	BPC2	BPC3	BPC4
K_SV_ (M^−1^)	157	176	147	152
Φ_et_(s1) ^a^	0.76	0.78	0.75	0.76

^a^ Φ_et_(s1) = K_sv_[Iod]/(1 + K_sv_[Iod]).

## Supplementary Materials

The following are available online at https://www.mdpi.com/2073-4360/12/6/1394/s1. Figure S1: Photolysis of (a) BPC2/TEOA; (b) BPC3/TEOA; (c) BPC4/TEOA; (d) C5/TEOA; (e) C7/TEOA upon LED@375 nm irradiation ([TEOA]=0.01M). Figure S2: (a) Fluorescence quenching of BPC2 by Iod in acetonitrile; (b) Stern–Volmer treatment for BPC2/Iod fluorescence quenching; (c) Fluorescence quenching of BPC3 by Iod in acetonitrile; (d) Stern–Volmer treatment for BPC3/Iod fluorescence quenching; (e) Fluorescence quenching of BPC4 by Iod in acetonitrile; (f) Stern–Volmer treatment for BPC4/Iod fluorescence quenching. Figure S3: (a) Fluorescence quenching of BPC1 by EDB in acetonitrile; (b) Fluorescence quenching of BPC2 by EDB in acetonitrile; (c) Fluorescence quenching of BPC3 by EDB in acetonitrile; (d) Fluorescence quenching of BPC4 by EDB in acetonitrile. Figure S4: Photopolymerization profile of TMPTA (acrylate function conversion vs. irradiation time) in laminate upon LED@405 nm irradiation in the presence of BPC1/EDB/Iod (0.5%/1%/1%, *w/w/w*). Synthetic procedure for the different derivatives.
