# Supplementary material for: Monocomponent Photoinitiators based on Benzophenone-Carbazole Structure for LED Photoinitiating Systems and Application on 3D Printing
Source: Polymers (Basel). 2020 Jun 22;12(6):1394. doi: 10.3390/polym12061394 (PMC7362224; doi:10.3390/polym12061394)
Supplement: Supplementary file 1 [file polymers-12-01394-s001.pdf]

Supporting Information

Monocomponent Photoinitiators Based on Benzophenone-Carbazole Structure for LED Photoinitiating Systems and Application on 3D Printing

Shaohui Liu[1,2], Hong Chen[1,2], Yijun Zhang[1,2], Ke Sun[1,2], Yangyang Xu[1,2], Fabrice Morlet-Savary[1,2], Bernadette Graff[1,2], Guillaume Noirbent[3], Corentin Pigot[3], Damien Brunel[3], Malek Nechab[3], Didier Gigmes[3], Pu Xiao[4]*, Frédéric Dumur[3]*, Jacques Lalevée[1,2]*

[1] Université de Haute-Alsace, CNRS, IS2M UMR 7361, F-68100 Mulhouse, France

[2] Université de Strasbourg, France

shaohui.liu@uha.fr (S.L.) ; hong.chen@uha.fr (H.C.) ; yijun.zhang@uha.fr (Y.Z.) ; ke.sun@uha.fr (K.S.) ; ahutxyy@163.com (Y.X.) ; fabrice.morlet-savary@uha.fr (F.M-S.); bernadette.graff@uha.fr (B.G.)

[3] Aix Marseille Univ, CNRS, ICR UMR 7273, F-13397 Marseille, France

[4] Research School of Chemistry, Australian National University, Canberra, ACT 2601, Australia

* Correspondence: pu.xiao@anu.edu.au; (P.X.); frederic.dumur@univ-amu.fr; (F.D.); jacques.lalevee@uha.fr; Tel.: +33 3 89 60 88 03 (J.L.)

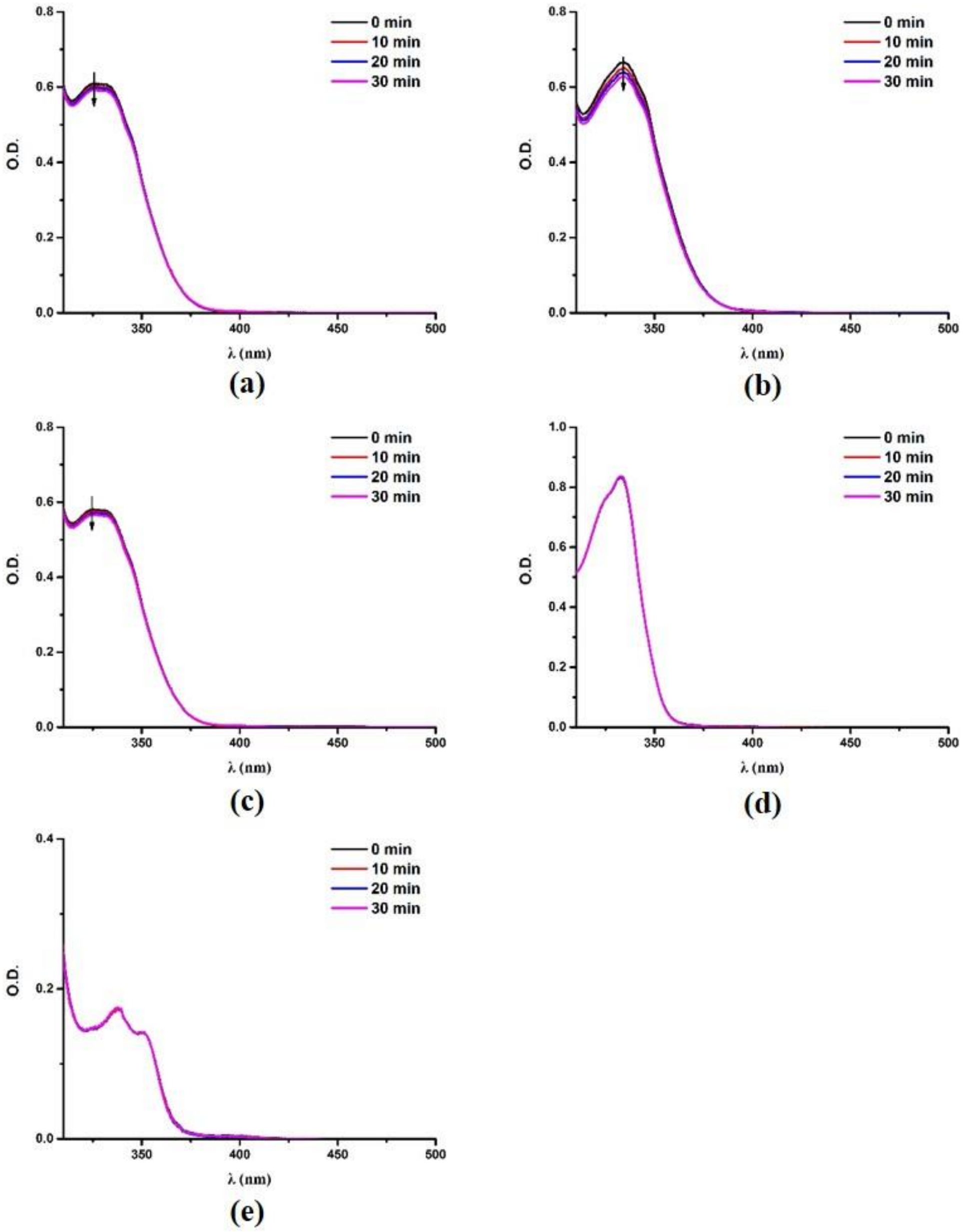

Figure S1. The photolysis of **(a)** BPC2/TEOA; **(b)** BPC3/TEOA; **(c)** BPC4/TEOA; **(d)** C5/TEOA; **(e)** C7/TEOA upon LED@375 nm irradiation ([TEOA]=0.01M).

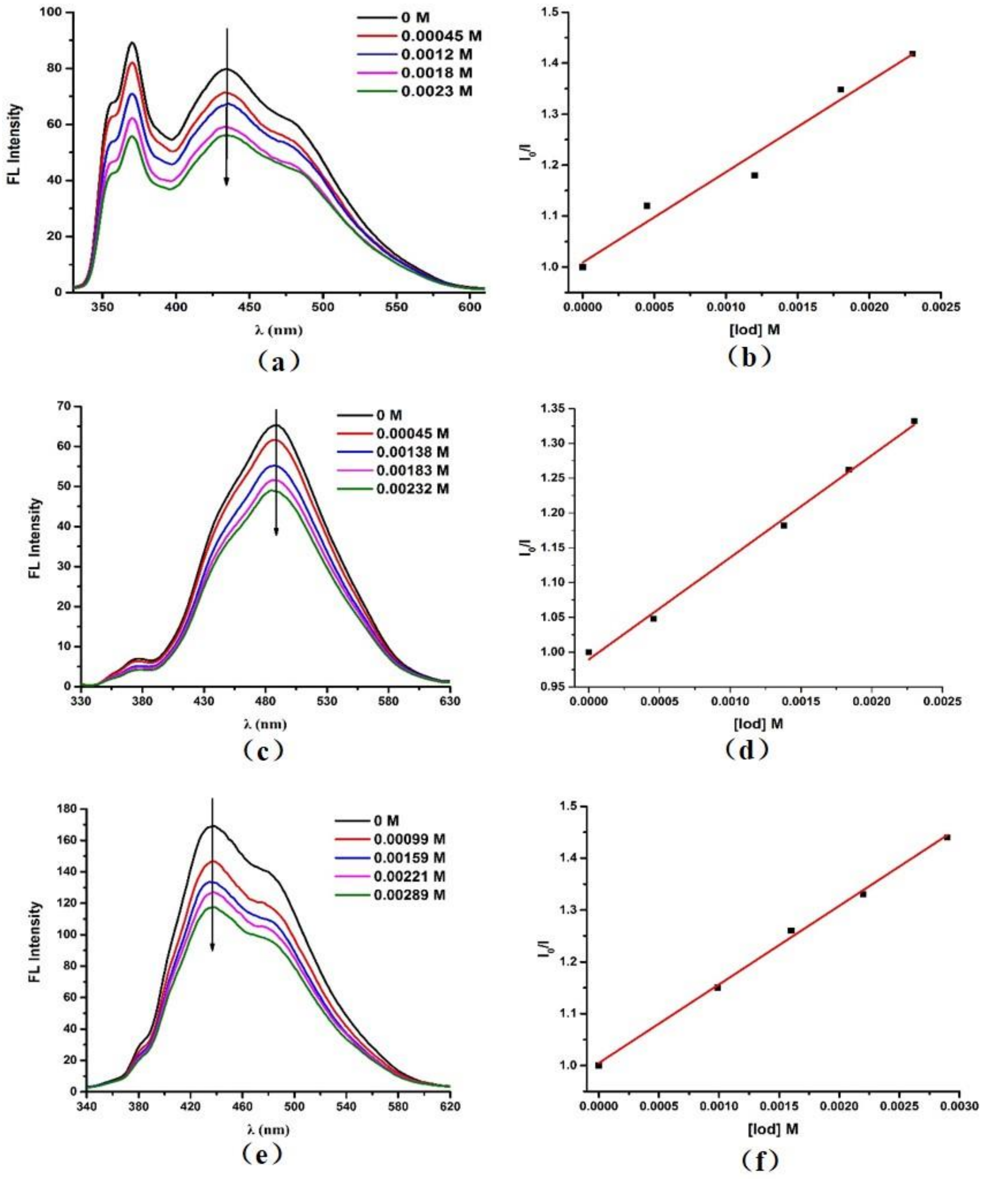

Figure S2. (a) Fluorescence quenching of BPC2 by Iod in acetonitrile; **(b)** Stern–Volmer treatment for BPC2/Iod fluorescence quenching; **(c)** Fluorescence quenching of BPC3 by Iod in acetonitrile; **(d)** Stern–Volmer treatment for BPC3/Iod fluorescence quenching; **(e)** Fluorescence quenching of BPC4 by Iod in acetonitrile; **(f)** Stern–Volmer treatment for BPC4/Iod fluorescence quenching.

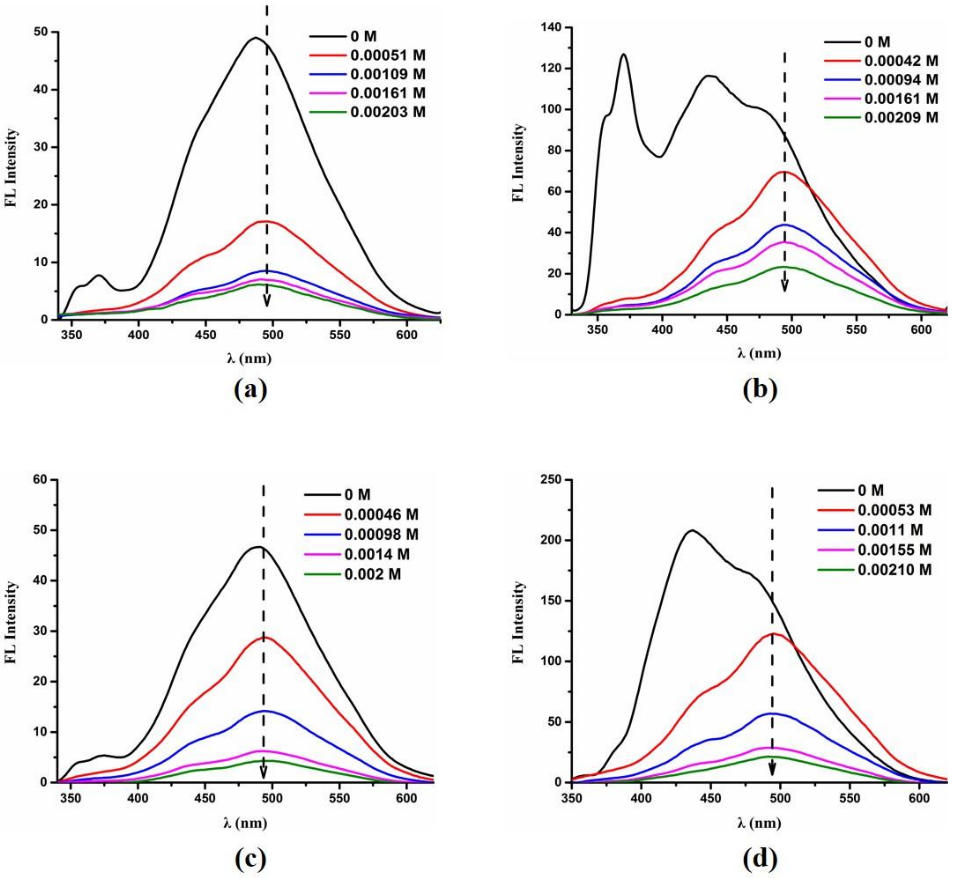

Figure S3. (a) Fluorescence quenching of BPC1 by EDB in acetonitrile; **(b)** Fluorescence quenching of BPC2 by EDB in acetonitrile; **(c)** Fluorescence quenching of BPC3 by EDB in acetonitrile; **(d)** Fluorescence quenching of BPC4 by EDB in acetonitrile.

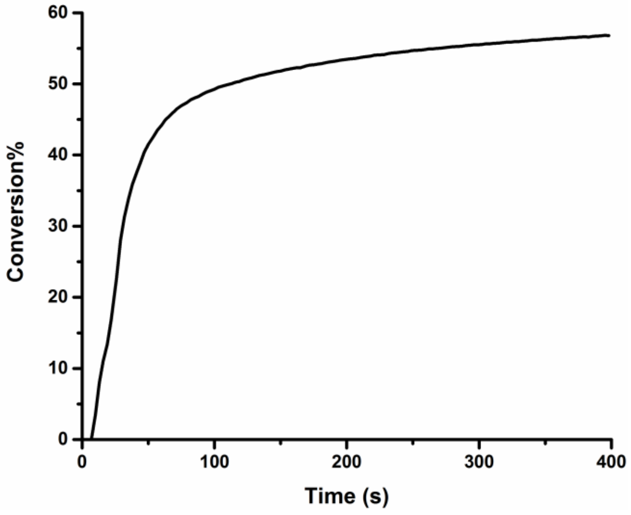

Figure S4. Photopolymerization profile of TMPTA (acrylate function conversion vs. irradiation time) in laminate upon LED@405 nm irradiation in the presence of BPC1/EDB/Iod (0.5%/1%/1%, w/w/w).

Experimental part

All reagents and solvents were purchased from Aldrich or Alfa Aesar and used as received without further purification. Mass spectroscopy was performed by the Spectropole of Aix-Marseille University. ESI mass spectral analyses were recorded with a 3200 QTRAP (Applied Biosystems SCIEX) mass spectrometer. The HRMS mass spectral analysis was performed with a QStar Elite (Applied Biosystems SCIEX) mass spectrometer. Elemental analyses were recorded with a Thermo Finnigan EA 1112 elemental analysis apparatus driven by the Eager 300 software. ^{1}H and ^{13}C NMR spectra were determined at room temperature in 5 mm o.d. tubes on a Bruker Avance 400 spectrometer of the Spectropole: ^{1}H (400 MHz) and ^{13}C (100 MHz). The ^{1}H chemical shifts were referenced to the solvent peak DMSO (2.49 ppm) and the ^{13}C chemical shifts were referenced to the solvent peak DMSO (49.5 ppm). All these carbazole photoinitiators were prepared with analytical purity up to accepted standards for new organic compounds (>98%) which was checked by high field NMR analysis.

Synthesis of (9-ethyl-9H-carbazol-3-yl)(phenyl)methanone ***BPC1***.

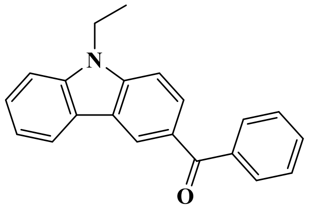

Chemical Formula: $C_{21}H_{17}NO$
Molecular Weight: 299.3730

$AlCl_3$ (3.60 g, 27 mmol, M = 133.34 g/mol) was added by portion to a cooled solution of 9-ethyl-9*H*-carbazole (5.27 g, 27 mmol, M = 195.26 g/mol) and benzoyl chloride (3.80 g, 3.13 mL, 27 mmol, M = 140.57 g/mol, d = 1.211) dissolved in dry dichloromethane (DCM) (25 mL, stabilized with amylene). The solution was stirred at room temperature overnight. The solution was poured into water. The aqueous phase was extracted several times with DCM. The organic phases were combined, dried over magnesium sulfate and the solvent removed under reduced pressure. The residue was

purified by column chromatography (SiO_2) using DCM as the eluent. ^{1}H NMR ($CDCl_3$) δ : 1.49 (t, 3H, J = 7.2 Hz), 4.42 (q, 2H, J = 7.2 Hz), 7.29 (td, 1H, J = 7.9 Hz, J = 1.0 Hz), 7.45-7.47 (m, 2H), 7.50-7.54 (m, 3H), 7.58-7.62 (m, 1H), 7.84-7.87 (m, 2H), 8.04 (dd, 1H, J = 8.6 Hz, J = 1.7 Hz), 8.12 (d, 1H, J = 7.7 Hz), 8.62 (d, 1H, J = 1.3 Hz); ^{13}C NMR ($CDCl_3$) δ : 13.8, 37.9, 108.0, 109.0, 120.0, 120.8, 122.6, 123.2, 124.1, 126.5, 128.2, 128.5, 128.6, 129.9, 131.6, 139.1, 140.7, 142.6, 196.6; HRMS (ESI MS) m/z: theor: 300.1383 found: 300.1384 ($[M+H]^+$ detected).

Synthesis of (9-ethyl-9H-carbazol-3-yl)(4-methoxyphenyl)methanone ***BPC2***

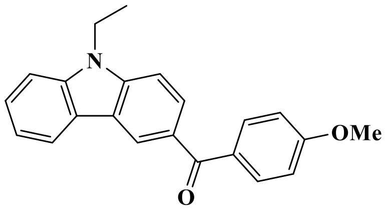

Chemical Formula: $C_{22}H_{19}NO_2$
Molecular Weight: 329.3990

$AlCl_3$ (3.60 g, 27 mmol, M = 133.34 g/mol) was added by portion to a cooled solution of 9-ethyl-9*H*-carbazole (5.27 g, 27 mmol, M = 195.26 g/mol) and 4-methoxybenzoyl chloride (4.60 g, 3.65 mL, 27 mmol, M = 170.59 g/mol, d = 1.260) was dissolved in dry DCM (25 mL, stabilized with amylene). The solution was stirred at room temperature overnight. The solution was poured into water. The aqueous phase was extracted several times with DCM. The organic phases were combined, dried over magnesium sulfate and the solvent removed under reduced pressure. The residue was purified by column chromatography (SiO_2) using DCM as the eluent. ^{1}H NMR ($CDCl_3$) δ : 1.48 (t, 3H, J = 6.6 Hz), 3.92 (s, 3H), 4.42 (q, 2H, J = 6.6 Hz), 7.01 (d, 2H, J = 8.8 Hz), 7.28 (td, 1H, J = 7.9 Hz, J = 1.0 Hz), 7.44-7.47 (m, 2H), 7.52 (td, 1H, J = 8.2 Hz, J = 1.0 Hz), 7.88 (d, 2H, J = 8.8 Hz), 8.00 (dd, 1H, J = 8.6 Hz, J = 1.7 Hz); ^{13}C NMR ($CDCl_3$) δ : 13.8, 37.8, 55.5, 107.9, 108.9, 113.5, 119.8, 120.8, 122.5, 123.2, 123.7, 126.4, 128.3, 129.1, 131.5, 132.4, 140.6, 142.3, 162.7, 195.6; HRMS (ESI MS) m/z: theor: 330.1489 found: 330.1485 ($[M+H]^+$ detected).

Synthesis of (9-dodecyl-9H-carbazol-3-yl)(phenyl)methanone ***BPC3***

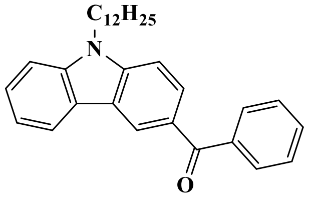

Chemical Formula: $C_{31}H_{37}NO$
Molecular Weight: 439.6430

$AlCl_3$ (3.60 g, 27 mmol, M = 133.34 g/mol) was added by portion to a cooled solution of 9-dodecyl-9*H*-carbazole (9.06 g, 27 mmol, M = 335.53 g/mol) and benzoyl chloride (3.80 g, 3.13 mL, 27 mmol, M = 140.57 g/mol, d = 1.211) dissolved in dry DCM (25 mL, stabilized with amylene). The solution was stirred at room temperature overnight. The solution was poured into water. The aqueous phase was extracted several times with DCM. The organic phases were combined, dried over magnesium sulfate and the solvent removed under reduced pressure. The residue was purified by column chromatography (SiO_2) using DCM as the eluent. ^{1}H NMR ($CDCl_3$) δ : 0.89 (t, 3H, J = 6.7 Hz), 1.20-1.45 (m, 20H), 1.91 (qt, 2H, J = 7.4 Hz), 4.34 (t, 2H, J = 7.2 Hz), 7.29 (td, 1H, J = 7.9 Hz, J = 0.9 Hz), 7.44-7.46 (m, 2H), 7.50-7.54 (m, 3H), 7.59-7.63 (m, 1H), 7.85-7.87 (m, 2H), 8.05 (dd, 1H, J = 8.6 Hz, J = 1.5 Hz), 8.11 (d, 1H, J = 7.7 Hz), 8.62 (d, 1H, J = 1.5 Hz); ^{13}C NMR ($CDCl_3$) δ : 14.1, 22.7, 27.3, 29.0, 29.3, 29.4, 29.5, 29.56, 29.60, 31.9, 43.4, 108.3, 109.2, 119.9, 120.7, 122.5, 123.2, 124.0, 126.4, 128.2, 128.4, 129.9, 131.6, 139.1, 141.2, 143.1, 196.6; HRMS (ESI MS) m/z: theor: 440.2948 found: 440.2942 ($[M+H]^+$ detected).

Synthesis of (9-dodecyl-9H-carbazol-3-yl)(4-methoxyphenyl)methanone ***BPC4***

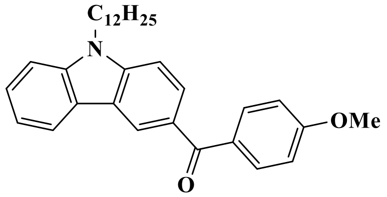

Chemical Formula: $C_{32}H_{39}NO_2$
Molecular Weight: 469.6690

$AlCl_3$ (3.60 g, 27 mmol, M = 133.34 g/mol) was added by portion to a cooled solution of 9-dodecyl-9*H*-carbazole (9.06 g, 27 mmol, M = 335.53 g/mol) and 4-methoxybenzoyl chloride (4.60 g, 3.65 mL, 27 mmol, M = 170.59 g/mol, d = 1.260)

was dissolved in dry DCM (25 mL, stabilized with amylene). The solution was stirred at room temperature overnight. The solution was poured into water. The aqueous phase was extracted several times with DCM. The organic phases were combined, dried over magnesium sulfate and the solvent removed under reduced pressure. The residue was purified by column chromatography (SiO_2) using DCM as the eluent. ^{1}H NMR ($CDCl_3$) δ : 0.89 (t, 3H, J = 6.7 Hz), 1.20-1.45 (m, 20H), 1.91 (qt, 2H, J = 7.4 Hz), 3.92 (s, 3H), 4.34 (t, 2H, J = 7.2 Hz), 7.01 (d, 2H, J = 7.9 Hz), 7.28 (t, 1H, J = 7.8 Hz), 7.43-7.46 (m, 2H), 7.51 (td, 1H, J = 7.0 Hz, J = 1.1 Hz), 7.88 (d, 2H, J = 7.9 Hz), 8.00 (dd, 1H, J = 8.6 Hz, J = 1.5 Hz), 8.11 (d, 1H, J = 7.7 Hz), 8.57 (d, 1H, J = 1.5 Hz); ^{13}C NMR ($CDCl_3$) δ : 14.1, 22.7, 29.0, 29.3, 29.4, 29.5, 29.55, 29.60, 31.9, 43.4, 55.5, 108.2, 109.2, 113.5, 119.8, 120.7, 122.4, 123.1, 123.6, 126.3, 128.2, 129.1, 131.5, 132.4, 141.1, 142.8, 162.7, 195.5; HRMS (ESI MS) m/z: theor: 470.3054 found: 470.3057 ($[M+H]^+$ detected).

Synthesis of 1,1'-(9-dodecyl-9H-carbazole-3,6-diyl)bis(ethan-1-one) ***C5***

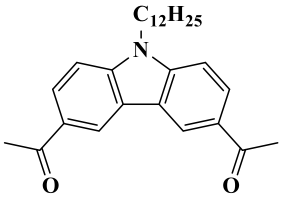

Chemical Formula: $C_{28}H_{37}NO_2$
Molecular Weight: 419.6090

$AlCl_3$ (3.60 g, 27 mmol, M = 133.34 g/mol) was suspended in dry DCM (25 mL, stabilized with amylene) and acetyl chloride (4.4 g, 3.84 mL, 54 mmol, M = 78.48 g/mol, d = 1.104) were added under ice-bath. The mixture was stirred for 15 min, after which 9-dodecyl-9*H*-carbazole (9 g, 27 mmol, M = 335.53 g/mol) was added. The solution was stirred at 0 ℃ overnight and quenched with water. The solution was extracted several times with DCM. The organic layers were combined, dried over magnesium sulfate and the solvent removed under reduced pressure. The residue was purified by filtration on a plug of silicagel using chloroform as the eluent. ^{1}H NMR ($CDCl_3$) δ : 0.86 (t, 3H, J= 6.7 Hz), 1.24-1.38 (m, 20H), 1.86 (qt, 2H, J = 4.5 Hz), 2.74 (s, 6H), 4.32 (t, 2H, J = 7.2 Hz), 7.45 (d, 2H, J = 8.6 Hz), 8.18 (dd, 2H, J = 8.6 Hz, J = 1.7 Hz), 8.79 (d, 2H, J = 1.7 Hz); ^{13}C NMR ($CDCl_3$) δ : 14.1, 22.7, 26.6, 27.2, 28.9, 29.3, 29.4, 29.50, 29.52, 29.56, 29.58, 31.8, 43.7, 109.0, 122.0, 122.9, 127.0, 129.8,

144.0, 197.4; HRMS (ESI MS) m/z: theor: 420.2897 found: 420.2899 ($[M+H]^+$ detected).

Synthesis of 1-(9-dodecyl-9H-carbazol-3-yl)ethan-1-one ***C6***

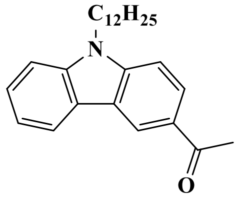

Chemical Formula: $C_{26}H_{35}NO$
Molecular Weight: 377.5720

$AlCl_3$ (3.60 g, 27 mmol, M = 133.34 g/mol) was suspended in dry DCM (25 mL, stabilized with amylene) and acetyl chloride (2.2 g, 1.92 mL, 27 mmol, M = 78.48 g/mol, d = 1.104) were added under ice-bath. The mixture was stirred for 15 min, after which 9-dodecyl-9*H*-carbazole (9 g, 27 mmol, M = 335.53 g/mol) was added. The solution was stirred at 0 ℃ overnight and quenched with water. The solution was extracted several times with DCM. The organic layers were combined, dried over magnesium sulfate and the solvent removed under reduced pressure. The residue was purified by filtration on a plug of silicagel using chloroform as the eluent. ^{1}H NMR ($CDCl_3$) δ : 0.88 (t, 3H, J = 6.7 Hz), 1.24-1.38 (m, 20H), 1.86 (qt, 2H, J = 4.5 Hz), 2.73 (s, 3H), 4.32 (t, 2H, J = 7.2 Hz), 7.30 (td, 1H, J = 7.9 Hz, J = 1.0 Hz), 7.41 (d, 1H, J = 8.6 Hz), 7.43-7.45 (m, 1H), 7.51 (t, 1H, J = 8.2 Hz), 8.13 (dd, 1H, J = 7.1 Hz, J = 1.2 Hz), 8.17-8.18 (m, 1H); ^{13}C NMR ($CDCl_3$) δ : 14.1, 22.7, 26.6, 27.2, 28.9, 29.3, 29.4, 29.6, 29.53, 29.58, 31.9, 43.4, 108.3, 109.2, 119.9, 120.6, 121.9, 122.6, 123.2, 126.40, 126.41, 128.8, 141.2, 143.2, 197.6; HRMS (ESI MS) m/z: theor: 377.2719 found: 377.2716 ($[M+H]^+$ detected).

Synthesis of 9-Ethyl-3-(1-phenylvinyl)-9H-carbazole ***C7***

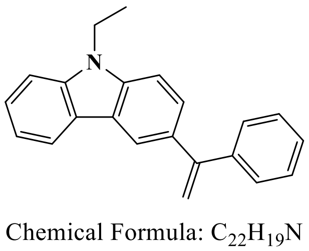

Chemical Formula: $C_{22}H_{19}N$
Molecular Weight: 297.4010

Magnesium (98%, 4.2 g, 170 mmol) and diethyl ether (20 mL) were stirred under nitrogen as iodomethane (99%, 25.0 g, 11 mL, 170 mmol, d = 2.28, M = 141.94 g/mol) was added dropwise over 20 min at such a rate as to maintain a continuous exotherm and gentle refluxing. The mixture was gently refluxed for 25 min by which time almost all of the magnesium had dissolved. The reaction mixture was protected from light and a solution of (9-ethyl-9*H*-carbazol-3-yl)(phenyl)methanone **BPC1** (9.89 g, 33 mmol, 1 eq., M = 299.37 g/mol) in toluene (280 mL) at room temperature was run in. The mixture was stirred under nitrogen in the absence of light at ambient temperature for 22 h, before cautiously quenching with water (200 mL) to give a light blue emulsion, which was stirred for 15 min. A solution of ammonium chloride (30 g) and acetic acid (99%, 15 mL) in water (150 mL) was added and the whole stirred for 3.5 h. The aqueous phase was washed twice with toluene (50 mL) and the extracts combined with the organic phase, from which the solvent was removed under reduced pressure. The residue was filtered on a plug of silicagel using DCM as the eluent. ^{1}H NMR ($CDCl_3$) δ : 1.32 (t, 3H, J = 7.2 Hz), 4.23 (q, 2H, J = 7.2 Hz), 5.39 (d, 1H, J = 1.3 Hz), 5.45 (d, 1H, J = 1.3 Hz), 7.07-7.17 (m, 2H), 7.24-7.31 (m, 4H), 7.34-7.40 (m, 4H), 7.96 (d, 1H, J = 7.7 Hz), 8.02 (d, 1H, J = 1.7 Hz); ^{13}C NMR ($CDCl_3$) δ : 13.8, 37.5, 107.9, 108.5, 113.0, 118.9, 120.2, 120.5, 122.8, 123.0, 125.3, 125.7, 126.3, 127.6, 128.1, 128.2, 128.4, 129.0, 132.5, 139.6, 140.3, 142.4, 150.7; HRMS (ESI MS) m/z: theor: 298.1590 found: 298.1585 ($[M+H]^+$ detected).

Synthesis of 9-ethyl-3-(1-(4-methoxyphenyl)vinyl)-9H-carbazole ***C8***

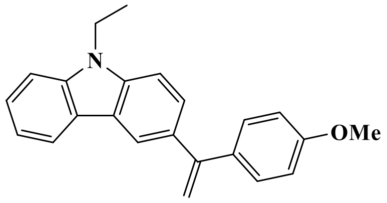

Chemical Formula: $C_{23}H_{21}NO$
Molecular Weight: 327.4270

Magnesium (98%, 4.2 g, 170 mmol) and diethyl ether (20 mL) were stirred under nitrogen as iodomethane (99%, 25.0 g, 11 mL, 170 mmol, d = 2.28, M = 141.94 g/mol) was added dropwise over 20 min at such a rate as to maintain a continuous exotherm and gentle refluxing. The mixture was gently refluxed for 25 min by which time almost

all of the magnesium had dissolved. The reaction mixture was protected from light and a solution of (9-ethyl-9*H*-carbazol-3-yl)(4-methoxyphenyl)methanone **BPC2** (10.87 g, 33 mmol, 1 eq., M = 329.40 g/mol) in toluene (280 mL) at room temperature was run in. The mixture was stirred under nitrogen in the absence of light at ambient temperature for 22 h, before cautiously quenching with water (200 mL) to give a light blue emulsion, which was stirred for 15 min. A solution of ammonium chloride (30 g) and acetic acid (99%, 15 mL) in water (150 mL) was added and the whole stirred for 3.5 h. The aqueous phase was washed twice with toluene (50 mL) and the extracts combined with the organic phase, from which the solvent was removed under reduced pressure. The residue was filtered on a plug of silicagel using DCM as the eluent. ^{1}H NMR ($CDCl_3$) δ : 1.34 (t, 3H, J = Hz), 3.73 (s, 3H), 4.24 (q, 2H, J = Hz), 5.32 (dd, 2H, J = 9.5 Hz, J = 1.4 Hz), 6.79 (d, 2H, J = 8.8 Hz), 7.06-7.09 (m, 2H), 7.14-7.19 (m, 2H), 7.27 (d, 2H, J = 8.8 Hz), 7.36 (td, 1H, J = 8.3 Hz, J = 1.4 Hz), 7.95 (d, 1H, J = 7.6 Hz), 7.98 (d, 1H, J = 1.3 Hz); ^{13}C NMR ($CDCl_3$) δ : 13.9, 37.6, 55.3, 108.0, 108.6, 111.8, 113.6, 118.9, 120.3, 120.6, 125.4, 125.7, 126.5, 128.3, 129.1, 129.6, 132.9, 135.0, 140.4, 150.3, 159.4; HRMS (ESI MS) m/z: theor: 327.1623 found: 327.1628 ($[M+H]^+$ detected).
